# Spatial Transmission Dynamics of Respiratory Syncytial Virus A in China

**DOI:** 10.1155/tbed/9926198

**Published:** 2025-11-19

**Authors:** Bin Yan, Jinfeng Wang, Chengdong Xu, Jun Liu

**Affiliations:** ^1^State Key Laboratory of Resources and Environmental Information System, Institute of Geographic Sciences and Natural Resources Research, Chinese Academy of Sciences, Beijing 100101, China; ^2^University of Chinese Academy of Sciences, Beijing 100049, China; ^3^Institute for Viral Disease Control and Prevention, Chinese Center for Disease Control and Prevention, Beijing 102206, China; ^4^National Key Laboratory of Intelligent Tracking and Forecasting for Infectious Diseases (NITFID), Institute for Viral Disease Control and Prevention, Chinese Center for Disease Control and Prevention, Beijing 102206, China

**Keywords:** drivers, phylogeography, respiratory syncytial virus, RSVA, spatial diffusion

## Abstract

Respiratory syncytial virus (RSV), a major cause of acute respiratory infections (ARIs) globally, poses a significant threat, especially to vulnerable populations. However, the spatial transmission dynamics of RSV strains, including the influence of environmental and socioeconomic factors, remain inadequately understood. This study applied genetic sequences and phylogenetic methods to quantify evolutionary and spatial dispersal dynamics of RSV subgroup A (RSVA) across China from 2011 to 2019. We assessed viral population trends, mapped interprovincial transmission patterns, and evaluated the influence of meteorological and socioeconomic factors on viral spread. Our results revealed cyclical fluctuations in effective population size every 3–5 years, and a predominant southward spread driven by interprovincial transmission networks. We found that higher winter relative humidity (RH), urbanization rate, and human mobility promoted viral spread, while higher winter temperature and elevated urban population density appeared to inhibit it. These findings provide crucial insights into RSVA dispersal in China, underscoring the importance of regional surveillance networks and targeted interventions to curb cross-regional spread, and offer a valuable framework to inform RSV vaccine rollout strategies and guide resource allocation in high-risk areas.

## 1. Introduction

Respiratory infectious diseases (RIDs) represent a growing global health burden, exacerbated by increasing global connectivity. As one of the most, respiratory syncytial virus (RSV), a prominent respiratory pathogen, disproportionately affects vulnerable populations, particularly children and the elderly. From 1995 to 2019, RSV was the leading cause of pediatric pneumonia worldwide [1]. Notably, RSV-associated pneumonia deaths among older adults aged 70 years and older increased significantly by 100.3% (92.4–108.6) [2]. Recent estimates for 2019 indicate that RSV was responsible for 33 million (25.4–44.6) cases of acute lower respiratory infections(ALRIs)in children under 5 years of age, leading to 3.6 million (2.9–4.6) hospitalizations and 26,300 (15,100–49,100) deaths from RSV-associated ALRI [3], with a higher risk in low- and middle-income countries. In China, surveillance data reveal that RSV is the most prevalent virus among acute respiratory infections (ARIs) in children under 5 years of age, with a detection rate of 25.7%, nearly twice that of the influenza virus (14.2%) [4].

RSV, also known as human RSV (HRSV) and human orthopneumovirus, is a negative-sense, single-stranded RNA virus belonging to the genus *Orthopneumovirus* of the family Pneumoviridae in the order Mononegavirales [5]. Its genome, approximately 15,200 nucleotides long, encodes 11 proteins, with the surface glycoproteins G and F serving as key antigens. RSV strains are classified into RSV subgroup A (RSVA) and B (RSVB) based on monoclonal antibody specificity to these glycoproteins. While the F protein is a primary target for vaccine and monoclonal antibody development, the G protein exhibits greater variability, featuring two hypervariable regions (HVRs) that are important markers for genotyping and evolutionary studies [6]. RSVA typically exhibits higher viral loads and faster transmission dynamics than RSVB [7], while both subtypes co-circulate, RSVA consistently dominates seasonal epidemics in China [4, 8]. Notably, RSVA is more strongly associated with severe clinical outcomes, including lower respiratory tract infections (LRTIs) in infants and young children [9].

While the seasonality, spatial distribution, and demographic characteristics of RSV incidence, along with shifts in predominant circulating genotypes in China, have been individually studied, a comprehensive understanding of RSV's evolutionary and transmission dynamics across China, and the interplay of factors influencing them, remains elusive [8, 10–12]. To address this critical gap, we compiled a unique dataset integrating socioeconomic and meteorological variables (e.g., population mobility and temperature) with the HVR2 of the RSVA G gene from various Chinese provinces. This study aimed to elucidate the phylogenetic characteristics and the driving forces behind the transmission of RSVA.

## 2. Materials and Methods

### 2.1. Genetic Data

In this study, we searched for RSVA sequences originating from China in GenBank using the keyword “Human respiratory syncytial virus A”. Meanwhile, the spatial and temporal information of the sequences, that is, their sampling time and location, was also summarized. Sequences were aligned against the NC_038235 reference strain, and those lacking precise collection dates or geographic locations were excluded from subsequent analyses. Due to the high genetic variability of the G gene, we focused our analysis on nucleotides 634–891, which correspond to the second HVR (HVR2) of the G protein coding sequence. Following extraction, we excluded sequences containing gaps or ambiguous bases. From the remaining Chinese sequences collected between 2011 and 2019 with complete provincial metadata, we performed spatiotemporal grouping by year and province, followed by removal of duplicate sequences within each group. This stringent filtering process yielded a dataset of 1081 RSVA G gene HVR2 sequences with verified spatiotemporal information.

**Figure 1:**
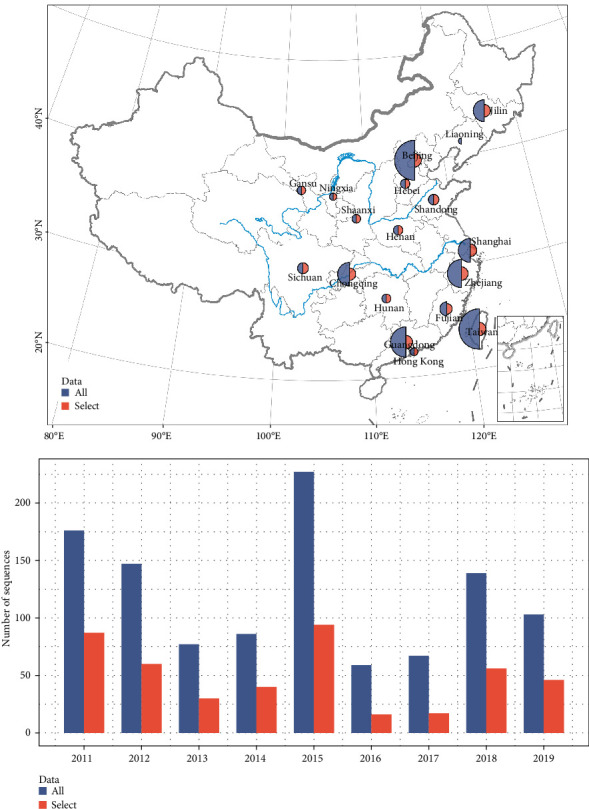
The spatial and temporal distribution of RSVA virus sequences. Blue (1081) shows the all data after pre-processing, and red (446) shows the data after downsampling selection. The size of the semicircle is proportional to numbers of viral sequences in the spatial distribution map.

To mitigate sampling bias associated with provincial population sizes and economic disparities, we implemented a stratified downsampling strategy. Sequences were first grouped by province of origin, and provinces containing fewer than five sequences were excluded. For remaining provinces, we applied the following sampling protocol: (1) provinces with 5–30 sequences were retained in their entirety; (2) provinces with 30–50 sequences were subsampled to 25 sequences; (3) provinces with 50–70 sequences to 30 sequences; (4) provinces with 70–100 sequences to 35 sequences; (5) provinces with >100 sequences to 40 sequences. Following comprehensive quality control, including gene extraction, deduplication, and downsampling, we obtained a final dataset of 446 high-quality sequences spanning 17 Chinese provinces for phylogenetic reconstruction (Figure 1). For generalized linear model (GLM) analysis, we further restricted the dataset to mainland China (395 sequences from 15 provinces) to match the available population flows data.

### 2.2. Covariate Data

Meteorological data, including average temperature (AT), precipitation (PP), relative humidity (RH), air pressure (AP), and wind speed (WS), were obtained from the China Integrated Meteorological Information Service System (https://data.cma.cn). The station data were interpolated to China using the Kriging method, and then the daily data for each province were averaged to obtain the winter mean meteorological data for each province. China's normalized difference vegetation index (NDVI) and night light data were downloaded from the resource and environmental data cloud platform (http://www.resdc.cn), which were also averaged and summarized by province. Annual provincial socioeconomic data, such as urbanization rate, urban population density, and GDP, were obtained from the National Bureau of Statistics (https://data.stats.gov.cn). These data are harmonized using 2015 values. The population flow data in this study were sourced from the 2023 provincial migration ratios provided by Baidu population migration numbers (https://qianxi.baidu.com). Specifically, the provincial population migration ratios calculated by aggregating real-world inter-city mobility patterns, represent the proportion of inter-provincial migrants relative to the national total, offering a robust proxy for actual population movement dynamics. The provincial migration data were structured into an origin-destination matrix using R (v4.2), where rows denote source provinces, columns indicate destination provinces, and cell values reflect the corresponding migration ratios.

### 2.3. Phylogenetic and Evolutionary Dynamic Analysis

Sequence alignment was performed using MAFFT [13, 14], followed by extraction of the 634–891 nucleotide segment corresponding to the HVR2 of the G gene coding sequence, using NC_038235 as the reference genome. We next generated a maximum likelihood (ML) phylogenetic tree in IQ-TREE [15] and assessed its temporal signal strength with TempEst [16]. The GTR + F + G4 nucleotide substitution model was selected using ModelFinder [17] based on the best-fit criteria. The best-fit molecular clock model and tree prior were determined through marginal likelihood estimation using both path-sampling (ps) and stepping-stone (ss) approaches in BEAST [18]. A relaxed molecular clock with lognormal distribution [19] was implemented, along with a coalescent skygrid prior to model temporal changes in effective population size [20]. Parameters, such as tMRCA and evolutionary rate were estimated using the BEAST package (version 1.10.4) [18]. Two independent chains were run with a chain length of 2 × 10^8^, and then Tracer (v1.7) [21] for convergence and mixture analysis with a burn-in period of 10% of the total chain length, with an effective sampling size >200 guaranteed for all parameters. Bayesian SkyGrid was used as the tree prior to estimate the effective population size over time. MCC trees were generated using TreeAnnotator (version 1.10.4) and illustrated in ggtree [22].

### 2.4. Discrete Phylogeographic Analyses

We implemented an asymmetric discrete-trait phylogeographic model in BEAST, combined with Bayesian Stochastic Search Variable Selection (BSSVS), to reconstruct the spatiotemporal dissemination history of RSVA across Chinese provinces [23]. The Markov chain Monte Carlo (MCMC) method was implemented with two independent chains, each comprising 2 × 10^8^ steps and a sampling interval of 20,000 steps. The sampled steps were then combined across chains, with the initial 10% discarded as a result of the burn-in phase. The convergence of the MCMC in Tracer (v1.7) was verified to ensure that the effective sample size of parameters exceeded 200. Utilizing the posterior inference results from the BEAST analysis, the interprovincial viral migration patterns were quantified through the Markov jump counts between locations. The interprovincial migration routes were summarized using SpreaD3 [24], while the intensity of viral migration between provinces was calculated as the sum of incoming and outgoing migration events. Finally, map visualization was performed using the ggplot package in R (v4.2).

In addition, the GLMs extension of the discrete phylogeographic model [25] was used to jointly infer the relevance and contribution of potential predictors to the spatial diffusion patterns among discrete locations in BEAST. This approach models transition rates as a function of latent factors, and quantifies the degree of contribution and significance level of latent factors through the GLM coefficient and the Bayes factor. The Bayes factor represents the ratio of the posterior probability to the prior probability based on the explanatory factors entering the model. Generally, a Bayes factor greater than 3 and a posterior probability greater than 0.5 indicates that the data support the entry of the explanatory factors into the model in a suggestive way, if the Bayes factor is greater than 20, indicating that the data is strongly supportive, and it is greater than 100 that indicates decisive support [26].

Current evidence indicates that both environmental and socioeconomic factors influence RSV prevalence and transmission dynamics [10, 27–31]. To systematically assess these associations, we selected the variables for analysis by considering the influence of covariates on RSV prevalence and the interdependence of covariates. Specifically, our framework incorporated three environmental variables (mean winter temperature, PP, and RH), five socioeconomic indicators (urbanization rate, urban population density, GDP, population flows, and night light index), and one spatial distance indicator (Haversine spherical distance). All environmental variables were derived from winter seasonal averages to align with the primary RSV transmission period. To account for potential sampling bias, we developed parallel models with and without sample size as a covariate. These analyses were restricted to sequences from mainland China with complete spatiotemporal metadata and matched covariate data.

## 3. Results

### 3.1. Evolutionary and Phylogeographic Analysis

Root-to-tip regression analysis revealed a significant temporal signal in the RSVA sequences (Supporting Information 1: Figure S2, correlation coefficient = 0.76, *R*^2^ = 0.58), with no outliers observed. This robust correlation validates the application of molecular clock models for time-calibrated phylogenetic reconstruction. Parameter estimates for molecular clock models are presented in Table 1. The Bayesian SkyGrid tree prior coupled with an uncorrelated lognormal relaxed clock emerged as the optimal configuration, with marginal likelihoods of −8127.9 (path sampling) and −8205.9 (ss sampling) supporting this selection.

**Figure 2:**
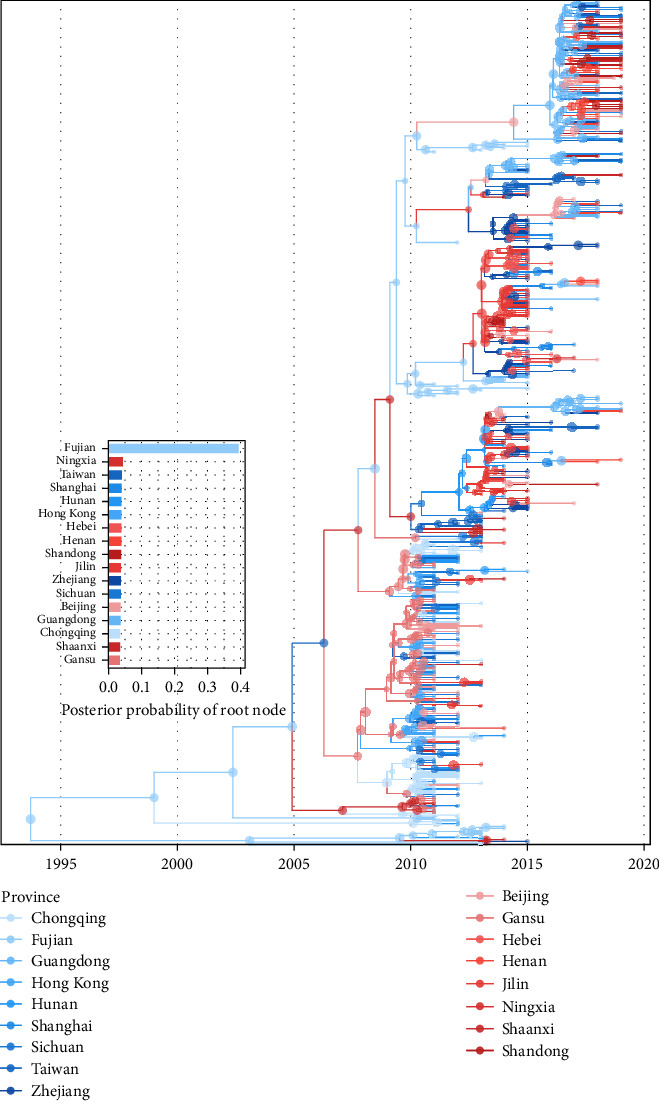
The maximum clade credibility tree of RSVA (446 sequences; 2011−2019). The phylogeny displays time-scaled branch lengths, with branch coloration indicating geographic origins. Southern provinces are represented in a blue gradient, while northern provinces are shown in a red gradient, with distinct color shades differentiating individual provinces. The embedded plot illustrates the posterior distribution of root node dates, using a color scheme that corresponds to the branches in the phylogenetic tree.

**Figure 3:**
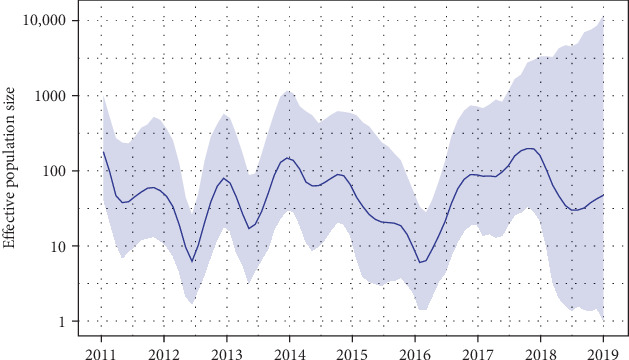
The effective population size through time. Solid lines represent the median estimates of effective population size and the shaded areas indicate the corresponding 95% credibility intervals.

The maximum clade credibility (MCC) tree (Figure 2) revealed the phylogenetic relationships of RSVA in China, in which the time of the most recent common ancestor (tMRCA) of RSVA was estimated to be 1993.5, with a 95% highest posterior density (HPD) range of 1976.7–2004.6, and the average evolutionary rate of the RSVA partial G gene was 5.27 (4.27–6.36) × 10^−3^ substitutions per site per year. The root location with the highest posterior probability was identified as Fujian, with a posterior probability of 0.39.  Figure 3 illustrates the dynamics of the effective population size of RSVA over time, as constructed by the Bayesian SkyGrid model. This construction indicates cyclical fluctuations with a period of 3–5 years between 2011 and 2019, marked by peaks in 2011, 2014, and 2018, and troughs in 2012 and 2016.

**Figure 4:**
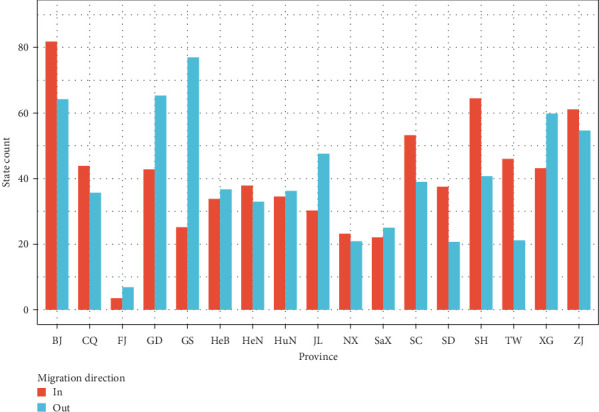
The histogram of state transition counts for locations. BJ: Beijing, CQ: Chongqing, FJ: Fujian, GD: Guangdong, GS: Gansu, HeB: Hebei, HeN: Henan, HuN: Hunan, JL: Jilin, NX: Ningxia, SaX: Shaanxi, SC: Sichuan, SD: Shandong, SH: Shanghai, TW: Taiwan, XG: Hong Kong, and ZJ: Zhejiang.

**Figure 5:**
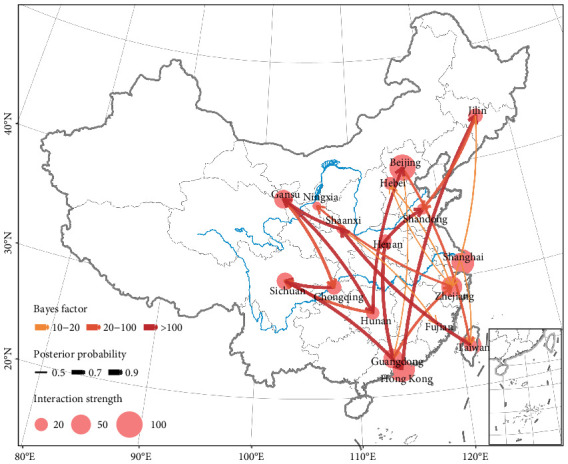
Spatial dispersal patterns of RSVA in China, determined by Bayesian phylogeography inference of G gene sequences. Curves show the between-province virus lineage transitions statistically supported with Bayes factor ≥ 3 and posterior probability ≥ 0.5. Pie represents the interaction strength among provinces by Markov jump counts.

To understand the dispersal of RSVA in China, we reconstructed the spatial transmission patterns of RSVA between 2011 and 2019.  Figure 4 shows the estimated migration counts of RSVA, indicating that Beijing, Shanghai, Zhejiang, Sichuan, Chongqing, Guangdong, Taiwan, and Hong Kong are major destinations, while Beijing, Guangdong, Hong Kong, Gansu, Zhejiang, Jilin, and Shanghai are significant sources, each with Markov jump counts exceeding 40, which confirms complex transmission dynamics.  Figure 5 illustrates the significant epidemiological unidirectional pathways of RSVA, supporting 21 migration paths by Bayesian phylogeographic analysis (BF ≥ 3 and posterior probability ≥ 0.5), with nine paths receiving decisive support (BF ≥ 100): Sichuan to Hong Kong, Guangdong to Henan, Taiwan to Shaanxi, Hong Kong to Jilin, Henan to Shandong, Chongqing to Sichuan, Shaanxi to Gansu, Gansu to Hunan, and Hunan to Beijing, and the remaining 12 paths with BF greater than 15. Within these paths, there were 10 transmission paths from north to south: Jilin to Shandong and Zhejiang, Hebei to Zhejiang, Beijing to Taiwan and Guangdong, Shanghai to Taiwan, Gansu to Chongqing and Hunan, Shaanxi to Fujian, and Sichuan to Hong Kong. Meanwhile, there were seven south-to-north transmission paths: Taiwan to Shaanxi, Hong Kong to Jilin, Guangdong to Zhejiang and Henan, Zhejiang to Hebei and Ningxia, and Hunan to Beijing. Also, we counted the location attributes of branches in the MCC tree (Figure 2) and found that the counts of location transitions for southward dispersal and northward dispersal are 103 and 88, respectively. We identified more significant paths for southward diffusion than for northward diffusion. Besides, the remaining paths spread between neighboring provinces, such as Chongqing to Sichuan, Henan to Shandong, and Shaanxi to Gansu.

### 3.2. Potential Factor Identification of Dispersal Patterns

**Figure 6:**
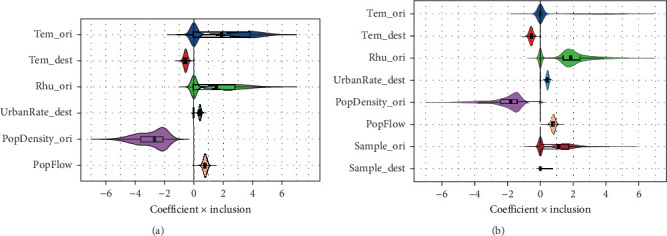
The contributions of significant predictor variables for RSVA dispersal. The *X*-axis represents the product of the coefficient and the inclusion probability for the predictors (coefficient × Inclusion). The gray boxes in the violin plots represent the median and quantile estimates. (a) and (b) distinguish between analyses without and with sample size predictors, respectively. The significant predictors include mean winter temperatures at the destination province (Tem_dest), winter relative humidity at the origin province (Rhu_ori), urbanization rate at the destination location (UrbanRate_dest), urban population density at the origin province (PopDensity_ori), and population flow (PopFlow).

Identifying covariates associated with RSVA spatial dispersal patterns in China is crucial for informing public health interventions. To quantify the contributions of potential drivers, we employed a GLM extension of Bayesian discrete phylogeographic inference to analyze interprovincial diffusion dynamics. Our analysis incorporated meteorological factors (temperature, PP, and RH), socioeconomic factors (the night light index, urbanization rate, population density, GDP, and population mobility), and spatial distances potentially influencing RSVA transmission. Using a comprehensive modeling approach that evaluated all predictors simultaneously, we identified several significant drivers of provincial-scale RSVA dispersal (Figure 6 and Supporting Information 1: Figure S1, Table 2). Notably, winter temperature, winter RH, urbanization rate, population density, and population mobility showed statistically significant associations with viral spread patterns.

The multivariable analysis revealed distinct directional effects: viral lineage diffusion was positively associated with mean winter RH in the origin province, urbanization rate in destination provinces, and population mobility between provinces. Conversely, negative associations were observed for the mean winter temperature in destination provinces and urban population density in origin provinces.

To assess potential sampling bias, we included sequence sample size as an additional covariate in sensitivity analyses. Notably, even when the sample size in the origin provinces was included as a significant predictor in the model (Figure 6 and Supporting Information 1: Figure S1, Table 2), the identified associations retained their consistency and stability.

## 4. Discussion

This study confirms that RSV was the predominant viral pathogen for ARI among children under 5 years of age in China between 2011 and 2019. This finding aligns with global observations of viral rebounds following the COVID-19 pandemic, where hospitalization rates in high-income regions have already returned to pre-epidemic levels by 2022 [32]. By integrating extensive genetic data of RSVA with natural, socioeconomic, and population flow data, as well as relevant temporal and geographic information, we reconstructed the dispersal patterns of RSVA within China. Our analysis revealed frequent interprovincial transmission events, characterized by a predominant north-to-south dispersal trend. Furthermore, we demonstrate that both environmental conditions and socioeconomic factors synergistically shape the spread dynamics of RSVA.

Bayesian phylogenetic analysis estimated the time to the most recent common ancestor (tMRCA) of RSVA strains isolated in China (2011–2019) to be 1993.5 (95% HPD: 1976.7–2004.6). Fujian province was identified as the most probable root location for RSVA transmission. This is supported by previous findings that Fujian Province harbored the highest number of co-circulating RSVA subtypes in China during 2011–2013 [8]. This observation underscores that Fujian province functions as a critical source of RSVA genetic diversity and a significant historical viral reservoir, even with limited observed gene flow.

The population dynamics of RSVA, characterized by 3–5 year fluctuations in effective population size between 2011 and 2019, with peaks in 2011, 2014, and 2018, and troughs in 2012 and 2016, which coincided with previous studies [8]. These fluctuations likely reflect the rapid turnover of dominant RSVA genotypes, particularly the swift replacement of the NA1 genotype by ON1, first identified in China in 2011 [8, 12]. The cyclical dynamics of the population are attributed to the continuous shift of major genotypes and subtypes associated with the alternating epidemics of RSVA and RSVB, which is related to the current immune profile of the population caused by the previously endemic strains, as well as the spatial spread of the virus across provinces and countries [33–35].

The migration analysis revealed distinct patterns of RSVA dissemination across China. Beijing, Shanghai, Zhejiang, Sichuan, and Taiwan were identified as key source provinces for viral importation, while Beijing, Guangdong, Hong Kong, Gansu, and Zhejiang served as major export hubs. The economically developed regions (notably Beijing and Shanghai) exhibited higher migration frequencies. Despite this, surveillance data indicated that nearly all provinces contributed to the overall spread of RSVA. Unlike the distance-dependent decay often observed in avian influenza transmission [36], our findings revealed complex spatial transmission patterns. Analysis of significant diffusion pathways (Bayes factor ≥ 3 and posterior probability ≥ 0.5) and the MCC tree consistently demonstrated a predominant southward dispersal trend, with viral lineages spreading more frequently from northern to southern provinces than vice versa. This directional bias likely reflects environmental influences, specifically the strong winter seasonality of northern epidemics. Lower winter temperatures in these regions may enhance RSV stability, potentially facilitating viral spread and accelerating evolutionary dynamics [11, 30].

Our study identifies winter temperature and RH as critical environmental determinants of RSVA transmission dynamics. The relationship between temperature and RSV infection exhibits a general U-shaped pattern, with peak activity occurring at temperatures above 24–30°C or within the 2–6°C range. Importantly, within colder climates, RSV transmission exhibits a negative correlation with temperature, an effect attributed to enhanced viral stability at lower temperatures [9, 37–39]. This finding is particularly relevant for China, where widespread low winter temperatures align with observations in colder climates; consequently, interprovincial RSV transmission shows a negative correlation with the mean winter temperature of destination provinces. In temperate and subtropical regions, RSV activity positively correlates with RH [9, 37–39]. For China, where winter RH generally falls within a moderate range, increasing humidity is associated with higher RSV transmission efficiency, leading to a positive correlation between interprovincial RSVA transmission and the winter RH of source provinces.

Socioeconomic factors, including population flows, urbanization rate, and urban population density, also significantly influence RSVA transmission. Population mobility directly contributes to the spatial spread of RSVA, consistent with recent evidence linking viral pedigree similarity to higher population migration flows [33]. Subsequently, destination urbanization rate positively influences viral spread, as economic development in urban centers enhances medical infrastructure, tourism appeal, and transportation networks, thereby increasing population influx and facilitating viral transmission through heightened social interactions in densely populated public spaces. Conversely, urban population density in source provinces exhibits a negative association with interprovincial viral dispersal. High-density provinces (e.g., Henan, Gansu, and Shaanxi) typically feature lower urbanization levels and are characterized by out-migration patterns dominated by young adults, which may potentially reduce viral export risk.

This study has several limitations. First, our analysis was constrained by the quality and completeness of publicly available RSVA genetic sequences. The reliance on while we endeavored to compile a comprehensive dataset, partial G gene sequences, and potential residual sampling biases, despite our mitigation efforts, may influence the reconstructed phylogenetic and dispersal patterns. Second, covariates were used from a single point in time, whereas genetic data spans many years, and the effect of those factors that change over time may be affected, for example, urbanization rate and population movement. Third, the significant effects of PP and RH on virus transmission were not consistently observed across all analyses. This may be due to the spatial heterogeneity of factors influencing RSV diffusion in China, as evidenced by differing correlations with humidity in northern and southern regions [10, 40]. Finally, RSV engages in complex interactions with other respiratory viruses, encompassing both synergistic and competitive relationships. Coinfections with influenza viruses, coronaviruses, parainfluenza viruses, adenoviruses, and other pathogens are associated with more severe clinical outcomes, such as asthma exacerbations or viral reassortment [41, 42]. Conversely, influenza viruses and RSV may exhibit a competitive interplay, where infection with one virus confers transient cross-protection against the other, potentially delaying the epidemiological circulation of RSV [43]. Future research should aim to collect more comprehensive datasets and further investigate the nuanced roles of PP and RH on RSVA transmission across distinct epidemiological regions.

## 5. Conclusion

Between 2011 and 2019, RSVA transmission in China exhibited a predominant north-to-south dispersal pattern, with provinces experiencing high population mobility serving as key transmission hubs. Our findings indicate that higher winter temperatures and lower RH were associated with reduced transmission, while urbanization and population mobility promoted virus spread. Therefore, we recommend: (1) strengthening surveillance in regions with significant population movement during the period of high humidity; (2) developing predictive models that integrate climatic and mobility data; (3) implementing targeted control strategies focused on key transmission hubs.

## Figures and Tables

**Table 1:** Comparison of parameter estimates between molecular clock models and tree prior.

Molecular clock model	Coalescent tree prior	Log marginal likelihood (path sampling)	Log marginal likelihood (stepping stone sampling)
Strict clock	Constant size	−8321.17	−8393.4
Strict clock	Exponential growth	−8305.38	−8370.67
Strict clock	Logistic growth	−8192.12	−8259.85
Strict clock	Bayesian SkyGrid	−8181.13	−8247.67
Strict clock	Bayesian Skyline	−8210.19	−8288.27
Uncorrelated lognormal relaxed clock	Constant size	−8293.78	−8371.57
Uncorrelated lognormal relaxed clock	Exponential growth	−8288.03	−8354.2
Uncorrelated lognormal relaxed clock	Logistic growth	−8260.37	−8328.34
Uncorrelated lognormal relaxed clock	Bayesian SkyGrid	**−8127.92**	−8205.93
Uncorrelated lognormal relaxed clock	Bayesian Skyline	−8161.28	−8236.69

*Note*: The best-fitting model is indicated in bold.

**Table 2:** The GLM model results.

Drivers	Inclusion	Coefficients × indicators (95% HPD)
Tem_dest	0.97	−0.54 (−0.83, −0.30)
Rhu_ori	0.85	1.86 (0, 3.88)
UrbanRate_dest	0.89	0.40 (0, 0.56)
PopDensity_ori	0.98	−2.02 (−4.50, −0.78)
PopFlow	0.98	0.78 (0.53, 1.05)
Sample_ori	0.59	0.99 (−0, 2.46)

*Note*: The inclusion represents probability that the predictor was included in the model. The coefficients × indicators represents the product of the coefficient and the inclusion probability for the predictors, which is the robust effect of the covariate on virus transmission.

## Data Availability

All data supporting the findings of this study are openly accessible. Genetic sequences were acquired from GenBank (https://www.ncbi.nlm.nih.gov/genbank/), and accession numbers are listed in Supporting Information 2: Table S1. Covariate data used for the analysis are deposited in FigShare (https://doi.org/10.6084/m9.figshare.27151554.v1).

## Supporting Information

Supporting Information 1 Figure S1. The contributions of all predictor variables for RSVA dispersal without (a) and with (b) the sample. The X-axis represents the product of the coefficient and the inclusion probability for the predictors (coefficient × Inclusion). Tem: mean winter temperatures, Pre: mean winter precipitation, Rhu: winter relative humidity, UrbanRate: urbanization rate, PopDensity: urban population density, GDP: gross domestic product, Light: night light index, Distance: Haversine spherical distance, PopFlow: population migration numbers. Figure S2. Temporal signal tested in TempEst of RSVA.

Supporting Information 2 Table S1. Accession number of the virus sequences used.
