# Supplementary figures and images for: Spatial Transmission Dynamics of Respiratory Syncytial Virus A in China
Source: Transbound Emerg Dis. 2025 Nov 19;2025:9926198. doi: 10.1155/tbed/9926198 (PMC12657092; doi:10.1155/tbed/9926198)

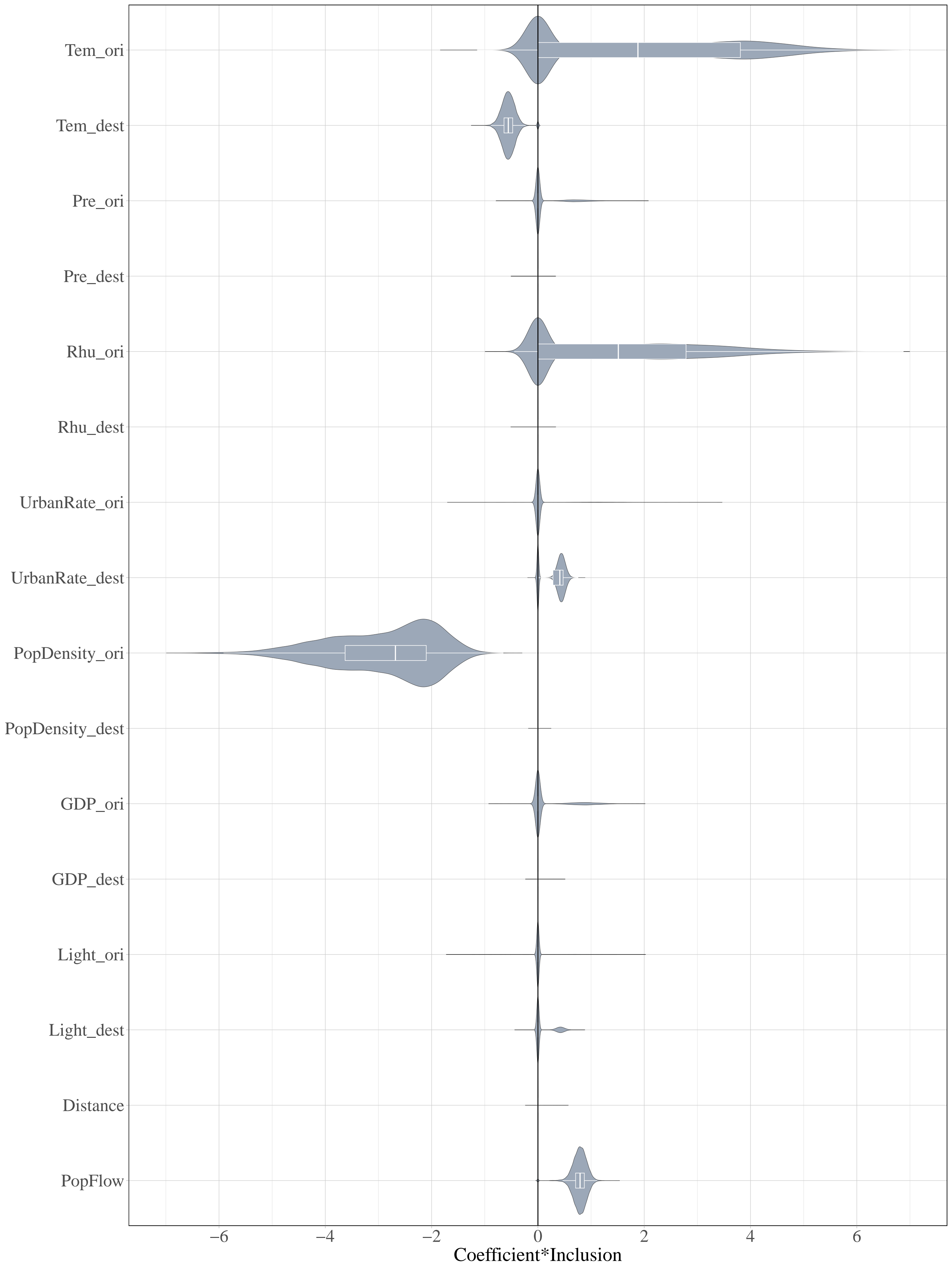

Supplement: Supporting Information 1 — Figure S1. The contributions of all predictor variables for RSVA dispersal without (a) and with (b) the sample. The X-axis represents the product of the coefficient and the inclusion probability for the predictors (coefficient × Inclusion). Tem: mean winter temperatures, Pre: mean winter precipitation, Rhu: winter relative humidity, UrbanRate: urbanization rate, PopDensity: urban population density, GDP: gross domestic product, Light: night light index, Distance: Haversine spherical distance, PopFlow: population migration numbers. Figure S2. Temporal signal tested in TempEst of RSVA. [file 9926198.f1.zip › 9926198.f1.zip/Figure S1.pdf]

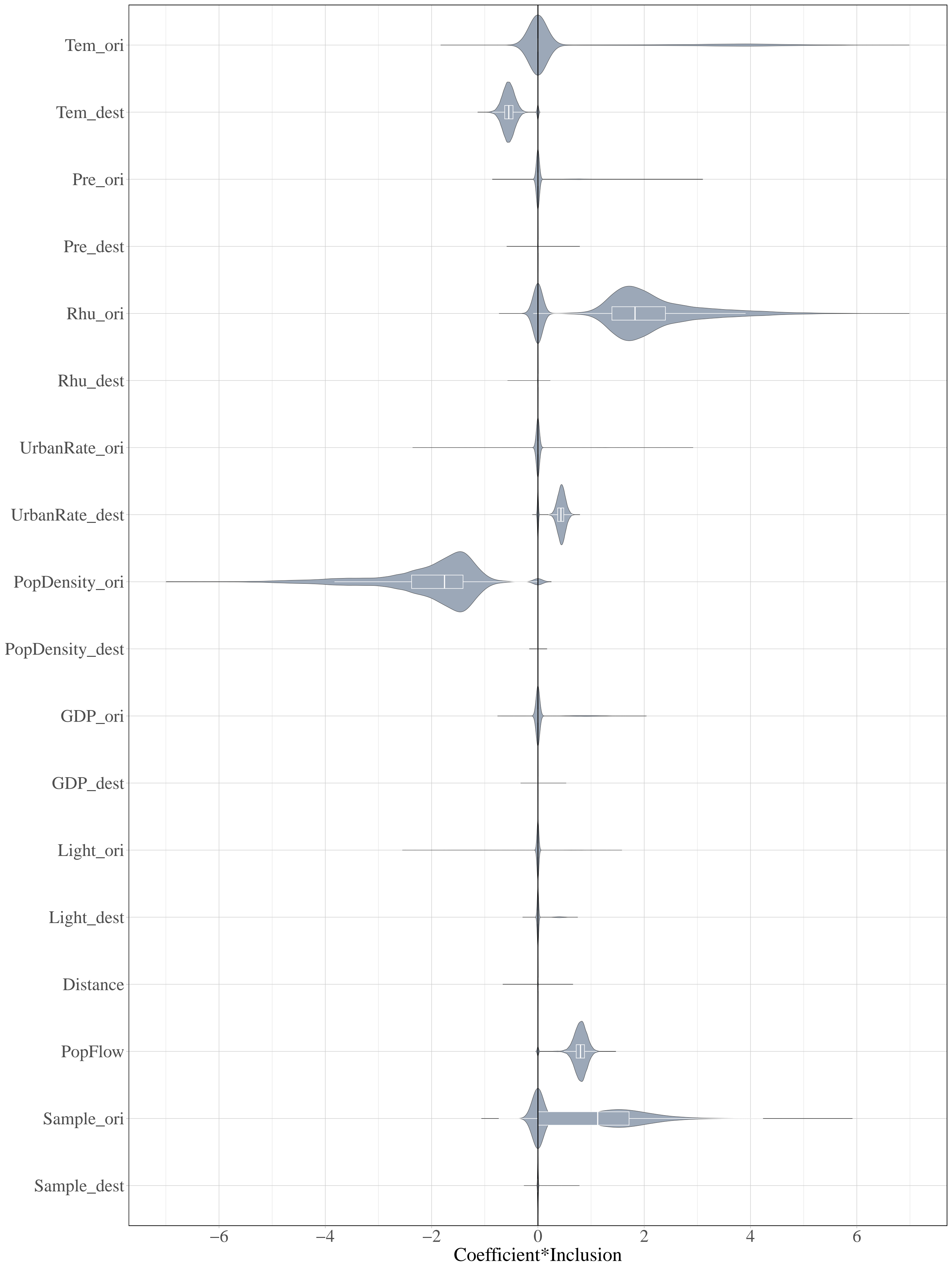

Supplement: Supporting Information 1 — Figure S1. The contributions of all predictor variables for RSVA dispersal without (a) and with (b) the sample. The X-axis represents the product of the coefficient and the inclusion probability for the predictors (coefficient × Inclusion). Tem: mean winter temperatures, Pre: mean winter precipitation, Rhu: winter relative humidity, UrbanRate: urbanization rate, PopDensity: urban population density, GDP: gross domestic product, Light: night light index, Distance: Haversine spherical distance, PopFlow: population migration numbers. Figure S2. Temporal signal tested in TempEst of RSVA. [file 9926198.f1.zip › 9926198.f1.zip/Figure S1b.pdf]

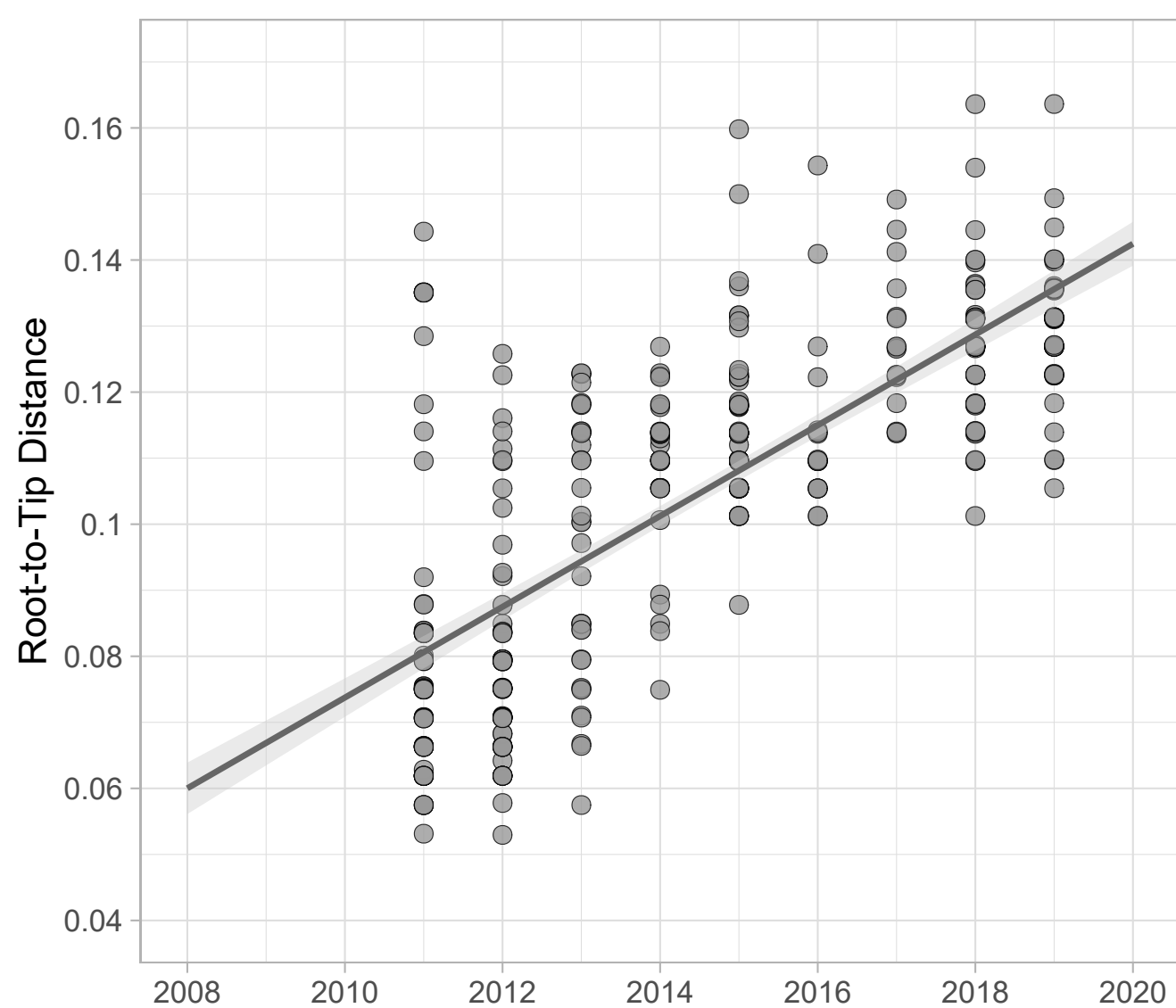

Supplement: Supporting Information 1 — Figure S1. The contributions of all predictor variables for RSVA dispersal without (a) and with (b) the sample. The X-axis represents the product of the coefficient and the inclusion probability for the predictors (coefficient × Inclusion). Tem: mean winter temperatures, Pre: mean winter precipitation, Rhu: winter relative humidity, UrbanRate: urbanization rate, PopDensity: urban population density, GDP: gross domestic product, Light: night light index, Distance: Haversine spherical distance, PopFlow: population migration numbers. Figure S2. Temporal signal tested in TempEst of RSVA. [file 9926198.f1.zip › 9926198.f1.zip/Figure S2.pdf]
